# Predictive Value of Serial Model of End-Stage Liver Disease Score Determination in Patients with Postcardiotomy Extracorporeal Membrane Oxygenation

**DOI:** 10.3390/jcm13071856

**Published:** 2024-03-23

**Authors:** Oliver Sommerfeld, Caroline Neumann, Marcel-Dominic Pfeifer, Gloria Faerber, Hristo Kirov, Christian von Loeffelholz, Torsten Doenst, Christoph Sponholz

**Affiliations:** 1 Department of Anaesthesiology and Critical Care Medicine, Jena University Hospital, Friedrich Schiller University Jena, 07747 Jena, Germany; caroline.neumann@med.uni-jena.de (C.N.); pfeifer.privat@gmx.de (M.-D.P.); christian.von_loeffelholz@med.uni-jena.de (C.v.L.); christoph.sponholz@med.uni-jena.de (C.S.); 2 Clinic for Cardiothoracic Surgery, Jena University Hospital, Friedrich Schiller University Jena, 07747 Jena, Germany; gloria.faerber@krz.uni-jena.de (G.F.); hristo.kirov@med.uni-jena.de (H.K.); torsten.doenst@med.uni-jena.de (T.D.)

**Keywords:** ECMO, multiple organ failure, MELD

## Abstract

(1) **Background**: The use of extracorporeal membrane oxygenation (ECMO) in low cardiac output states after cardiac surgery may aid in patient recovery. However, in some patients, the clinical state may worsen, resulting in multiple organ failure and high mortality rates. In these circumstances, calculating a model of end-stage liver disease (MELD) score was shown to determine organ dysfunction and predicting mortality. (2) **Methods**: We evaluated whether serial MELD score determination increases mortality prediction in patients with postcardiotomy ECMO support. (3) **Results**: Statistically, a cutoff of a 2.5 MELD score increase within 48 h of ECMO initiation revealed an AUC of 0.722. Further, we found a significant association between hospital mortality and 48 h MELD increase (HR: 2.5, 95% CI: 1.33–4.75, *p* = 0.005) after adjustment for possible confounders. (4) **Conclusions**: Therefore, serial MELD score determinations on alternate days may be superior to single measurements in this special patient cohort.

## 1. Introduction

Cardiogenic shock before or after cardiac surgery is associated with high mortality rates [1]. Besides the use of different vasoactive and inotropic substances, mechanical circulatory support by veno-arterial extracorporeal membrane oxygenation (va-ECMO) may aid patients in recovery, in transplantation, or in the insertion of permanent mechanical devices (LVAD). However, there is significant morbidity associated with this intervention, including neurologic complications, bleeding, and acute kidney injury [2]. It is therefore of interest to identify patients not responding to medical and mechanical support as early as possible.

In recent years, numerous scoring systems have been established to estimate the prognosis of patients on mechanical support; one of these is the survival after ECMO (SAVE) score, developed with the international Extracorporeal Life Support Organization (ELSO) [3]. However, given the complexity of calculating the SAVE score, evaluating more simple predictors of survival in these patients should be of interest.

In this respect, the model of end-stage liver disease (MELD) score may represent a tool to predict outcomes in this patient population. The MELD score was originally designed to predict mortality rates in patients with end-stage liver disease and was incorporated into the listing procedure for liver transplantation [4]. The score itself incorporates laboratory markers representing (1) the liver and (2) kidney function as well as (3) the coagulation system. Bilirubin and creatinine, two components of the MELD score, were independently shown to be predictive of mortality rates in patients undergoing va-ECMO therapy [5,6]. Especially kidney dysfunction (represented by acute kidney injury and/or need for renal replacement therapy) was found to be associated with morbidity and mortality in this critically ill patient cohort [7,8,9]. Additionally, liver dysfunction was shown to be associated with in-hospital mortality in patients supported with ECMO [10]. It is therefore not surprising that the results of both markers represent various predictive scores in ECMO patients [11] and may support calculating the MELD score in the present patient cohort.

In recent years, modifications of the MELD score have been able to predict patient survival in various clinical settings, including interventional and surgical cardiac scenarios [12,13,14]. With special focus on va-ECMO support, the MELD score has been able to predict survival with high accuracy, when calculated within 48 h of ECMO initiation [15]. However, as organ function may worsen or resolve with va-ECMO support, we suggest that serial MELD determination may have better prediction outcomes compared to a single value. The aim of our study was therefore to evaluate hospital mortality in va-ECMO patients based on a serial MELD score determination in a large patient cohort.

## 2. Materials and Methods

Patients were identified by charts from the Department of Cardiothoracic Surgery at Jena University Hospital. Patient characteristics, comorbidities, clinical scores, and laboratory results were taken from electronic patient charts (COPRA, version 6.78.2.0 and 5.24.974; COPRA System GmbH, Sasbachwalden, Germany) and the clinical database (SAP, version 7300.1.3.1079) on hospital admission, prior to ECMO initiation, as well as 24 h, 48 h, 72 h, and 96 h after ECMO initiation.

The study was approved by the ethical committee of Friedrich-Schiller University Jena, Germany (registration number: 2021-2503-Daten, Chairperson: Prof. E. Schleussner), on 4 January 2022. Informed consent was waived because of the anonymous and observational character of the study.

The MELD, CTP, and GFR scores were calculated from the obtained laboratory results. A Kaplan–Meier plot was generated to estimate survival, and a log-rank test was performed to compare survival curves. Multivariable analysis was performed using Cox’s proportional hazards model to determine the relationship between MELD score and hospital mortality. Models were adjusted for age, sex, and comorbidities. Receiver operating characteristic (ROC) curves were generated, and the area under the curve (AUC) was used to quantify the accuracy of MELD score in predicting hospital mortality. ROC curves and AUCs were also generated for the SAVE score. The Youden index analysis was employed to identify the optimal MELD score cutoff value.

Continuous data are presented in median [25th–75th percentile] values, and categorical data are displayed as numbers and percentages. Statistical analyses were performed using IBM SPSS Statistics, Version 26.0 (IBM Corporation, Armonk, NY, USA).

## 3. Results

### 3.1. Patient Characteristics and Laboratory Markers

Between 2010 and 2020, *n* = 13.304 patients underwent major cardiac surgery in our institution. Thereof, *n* = 338 (2.5%) patients were placed on va-ECMO support due to various reasons. Among them, *n* = 189 (1.4%) patients underwent perioperative va-ECMO support. For this evaluation, *n* = 77 patients were excluded because of an ECMO runtime of below 48 h (*n* = 73) or missing laboratory values for calculating MELD scores (*n* = 4). The remaining *n* = 112 (0.8%) patients had a median age of 69 [61.7–74.7] years and the majority were male (*n* = 81 [72.3%]). Most patients presented with a history of cardiovascular comorbidities (i.e., hypertension, coronary artery disease, or congestive heart failure). Patients (*n* = 112) had a median SAVE score of −9 [−15.0–−5.0] prior to va-ECMO initiation. The logistic EuroSCORE was only available in *n* = 109 patients with a median value of 7.15 [0.88–71.82]. The majority of patients (61%) required surgical re-exploration within the time period of va-ECMO treatment, most notably due to intrathoracic bleeding or cardiac tamponade. If necessary, re-exploration was undertaken in patients within 24 h after va-ECMO initiation. All patient characteristics are listed in Table 1.

After va-ECMO initiation, flow rates were anticipated at 2.5 L/m^2^. After va-ECMO initiation, laboratory results for bilirubin and creatinine, as well as MELD scores, moderately increased, whereas the glomerular filtration rate (GFR) decreased 24 h and 48 h after va-ECMO initiation when compared to levels at hospital admission and at initial va-ECMO initiation. For INR and CTP, only minor changes were found within this time period. The detailed ECMO flow rates, laboratory values, and MELD and CTP scores are listed in Table 2. 

### 3.2. Model of End-Stage Liver Disease Scores

**Figure 1:**
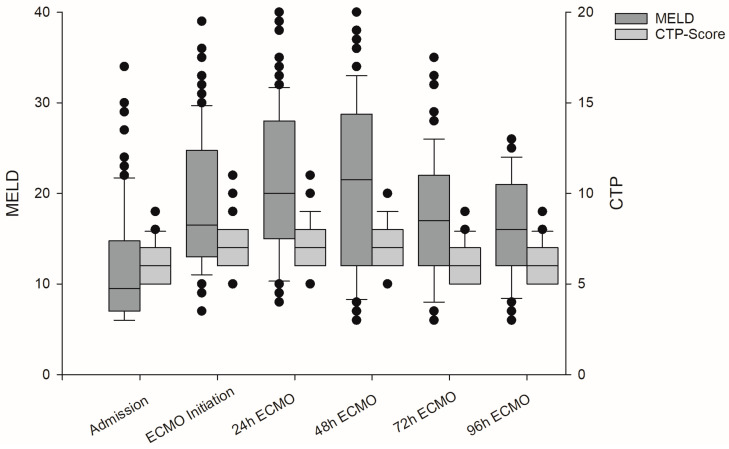
Calculated model of end-stage liver disease (MELD) and Child–Turcotte–Pugh (CTP) scores of the patient cohort at several time points: hospital admission, va-ECMO initiation, and 24, 48, 72, and 96 h after va-ECMO initiation.

Patients had a median MELD score of 10 [7.0–14.8] at hospital admission. MELD scores increased prior to ECMO initiation to 17 [13.0–24.8]. At 24 h and 48 h after ECMO initiation, MELD scores further increased to 20 [15.0–28.0] and 22 [12.0–28.8], respectively, and the declined at 72 h and 96 h after va-ECMO initiation. The median increase in MELD score was 2 [−2.0–+5.0] at 24 h (deltaMELD_24) and 1 [−3.0–+6.8] at 48 h (deltaMELD_48) compared to the score at initial ECMO initiation. In general, MELD score did not significantly increase at 48 h after ECMO initiation in comparison to at 24 h (deltaMELD_24-48: 0 [−3.0–+2.0]). In contrast, Child–Turcotte–Pugh (CTP) scores on average increased by one point after ECMO initiation and declined 48 h after ECMO initiation to the initial level; see Figure 1 and Table 2.

**Figure 2:**
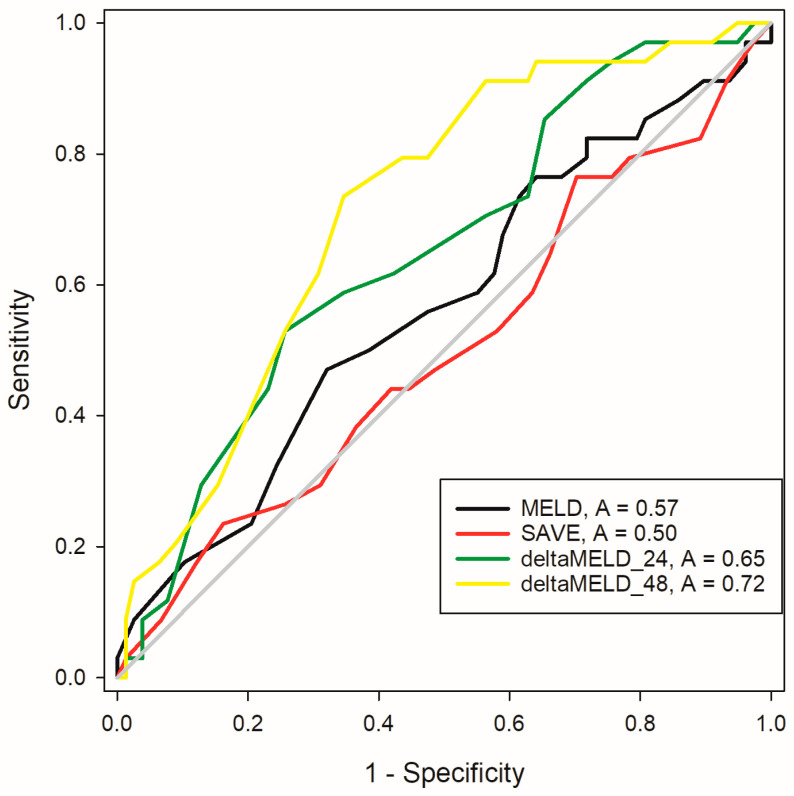
ROC curve analysis for MELD score, MELD score increases at 24 h (deltaMELD_24) and 48 h (deltaMELD_48) after va-ECMO, and SAVE score.

**Figure 3:**
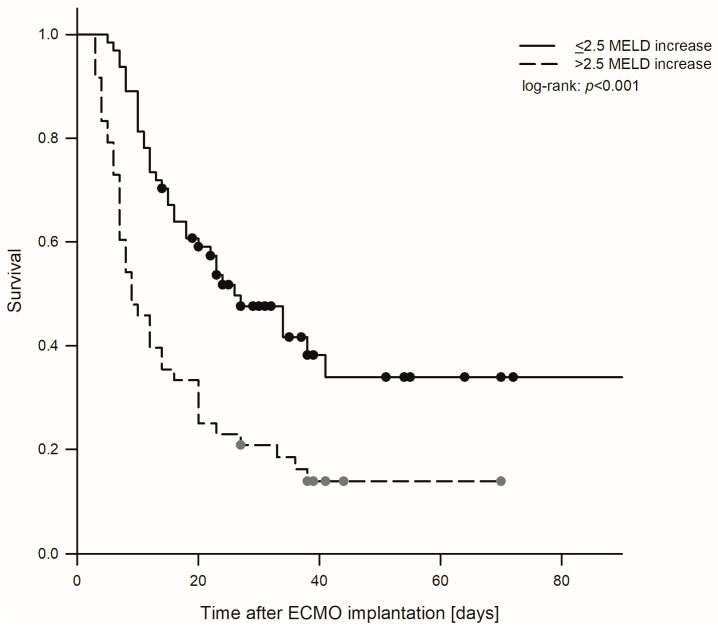
Kaplan–Meier estimates for hospital survival, comparing patients with MELD score increases of <2.5 or >2.5 points at 48 h after ECMO initiation.

ROC curve analysis revealed the highest AUC for deltaMELD_48 (0.722) followed by deltaMELD_24 (0.655) and MELD_ECMO initiation (0.571). The AUC of the SAVE score was 0.477 (Figure 2). The Youden index analysis demonstrated a MELD score increase of 2.5 as the optimal cutoff to limit risk. The resulting Kaplan–Meier analysis is shown in Figure 3. Cox’s proportional hazard analysis demonstrated that a deltaMELD_48 increase of >2.5 was predictive of hospital mortality. After adjustment for age, sex, BMI, surgical re-exploration, GFR, and creatinine after ECMO initiation and comorbidities, hospital mortality rate remained significantly higher for deltaMELD_48 (HR: 2.2, 95% CI: 1.31–3.66, *p* = 0.003).

## 4. Discussion

ECMO represents a therapeutic option for patients with postcardiotomy cardiogenic shock. However, cardiac function may not always recover and/or patient condition can deteriorate to multiple organ failure during the course of ECMO therapy. Therefore, markers and scores are warranted to easily identify deterioration with indicators regarding mortality. In this respect, Karnib and colleagues used MELD scores to predict 90-day mortality in patients undergoing ECMO therapy for cardiogenic shock. MELD scores were evaluated within 48 h of va-ECMO initiation and were predictive for 90-day mortality [15]. Nagy and colleagues were able to show MELD scores and MELD score modifications to be predictive in the risk stratification of va-ECMO in varying clinical indications [16], among them weaning failure from CPB. Compared to previous studies, the serial determination of the MELD score was used to analyze patients’ conditions in the current analysis. Here, MELD scores increased within the first 48 h of va-ECMO initiation and were predictive of mortality. Most likely due to limited ECMO runtime and/or the patient’s decease, the MELD score declined after 48 h. We therefore evaluated the course of MELD score within the first 48 h after ECMO initiation. Moreover, MELD score increases of >2.5 points within 48 h of va-ECMO initiation were predictive of hospital mortality, with an AUC under the ROC curve that was greater than the validated SAVE score or MELD score at initial ECMO. In contrast, CTP scores were not significantly altered within the time period and were therefore not analyzed.

Kidney function, represented by creatinine levels and GFR, is widely in use as an indicator to predict mortality in critically ill patients, among them patients on va-ECMO [7]. Both markers are routinely monitored in the ICU and are therefore easy to interpret. Likewise, liver dysfunction has also been shown to be predictive for ICU patients, especially those on va-ECMO [10]. A meta-analysis identified liver dysfunction to be associated with outcomes after cardiac surgery, using either MELD or the Child–Turcotte–Pugh scores for the definition of liver dysfunction [17]. As all of the MELD laboratory markers are included in clinical routines, this score is easy to calculate and frequently available. It is therefore not surprising that the score can be applied to various clinical conditions, including cardiac procedures [12,13,14]. Recently, risk stratification using the MELD score was used to be a predictor of in-hospital mortality in patients undergoing elective cardiac surgery. With a cutoff value of >20, patients had a hospital mortality rate of 31.2% [18]. In particular, progress over time may help to identify patients at risk and enhance the prediction model for va-ECMO patients.

ECMO support carries the risk of multiple complications associated with morbidity and mortality, among them the ischemia of the lower limbs, neurological events, acute kidney injury, bleeding, or infection [2,19]. In particular, bleeding complications were reported to be present in more than 40% of patients [2] and were the most reported complications after the initiation of postcardiotomy ECMO [19]. This finding is supported by current evaluations with the need for surgical re-exploration due to intrathoracic bleeding in around 40% of patients. Moreover, acute kidney injury and the revision of the femoral cannula to prevent lower limb ischemia were frequently reported in our patient cohort.

Multiple organ and persistent heart failure represent the leading causes of mortality in postcardiotomy ECMO patients [1,20]. This is also shown in the current analysis. As progression toward or away from multiple organ failure is a dynamic process, taking serial measurements of markers indicating organ function may early identify patients at risk, may provide possibilities of further treatment options, or may lead to limited therapy. Therefore, the current analysis does not reflect a self-fulfilling prophecy, as it shows that patients with high, but declining, MELD score at 48 h after ECMO therapy have a significant chance of survival in comparison to patients with an initial low, but increasing, MELD score, who are heading towards multiple organ failure.

## 5. Conclusions

Taken together, va-ECMO support may provide a therapeutic option for patients with postcardiotomy cardiogenic shock. However, due to the high rate of multiple organ failure as one of the leading causes of mortality in these patients, the early identification of patients at risk of mortality is warranted. In this respect, calculating the MELD score early after ECMO initiation might be a reliable marker to predict mortality. Serial determination on alternate days may enhance the validity of the MELD score in this special patient cohort, but further data are warranted to support this hypothesis.

## Figures and Tables

**Table 1:** Patient characteristics, va-ECMO indication, and revision and surgical re-exploration rate.

Variables	Total Cohort (*n* = 112)
Patient characteristics	
Age [years]	69 [61.7–74.7]
Male [n (%)]	81 (72.3)
BMI [kg/m^2^]	28 [24.9–31.0]
Comorbidities, n (%)	
CNS (Stroke/TIA)	5 (4.5)
Hypertension	99 (88.4)
CAD	81 (72.3)
STEMI	6 (5.4)
NSTEMI	22 (19.6)
Congestive heart failure	81 (72.3)
Diabetes	36 (32.1)
COPD	23 (20.5)
Cancer	1 (0.9)
Previous cardiac surgery	20 (17.9)
Log. EuroSCORE (patients *n* = 109; *n* = 3 missing data)	7.15 [0.88–71.82]
SAVEscore	−9 [−15.0–−5.0]
ECMO indication, *n* (%)	
CPB weaning failure, elective surgery	33 (29.5)
CPB weaning failure, urgent surgery	26 (23.2)
Postop. LCOS, elective surgery	35 (31.3)
Postop. LCOS, urgent surgery	18 (16.1)
Re-operation within 96 h of ECMO initiation, *n* (%)	
No re-operation	44 (39.3)
Bleeding or cardiac tamponade	45 (40.2)
Cardiac bypass revision	8 (7.1)
Revision of femoral cannulation	7 (6.3)
Laparotomy	7 (6.3)
ECMO revision	1 (0.9)
Re-operation time, *n* (%)	
No re-operation	44 (39.3)
Within 24 h of ECMO initiation	55 (49.1)
Within 24–48 h of ECMO initiation	4 (3.6)
More than 48 h after ECMO initiation	9 (8.0)

BMI: Body mass index; CNS: central nervous system; CAD: coronary artery disease; STEMI: ST-elevation infarction; NSTEMI: non-ST-elevation infarction; COPD: chronic obstructive pulmonary disease; CPB: cardiopulmonary bypass; Postop. LCOS: postoperative low cardiac output syndrome (within 6 h of surgery).

**Table 2:** Laboratory markers, clinical scores, and ECMO flow rates at several time points (MELD (model of end-stage liver disease) and CTP (Child–Turcotte–Pugh) scores).

	Hospital Admission	ECMO Initiation	24 h after ECMO Initiation	48 h after ECMO Initiation	72 h after ECMO Initiation	96 h after ECMO Initiation
Bilirubin (µmol/L)	14 [9.0–18.0]	25 [15.0–37.8]	34 [16.0–61.5]	37 [15.0–78.5]	38 [15.5–80.0]	38 [14.5–73.8]
Creatinine (µmol/L)	90 [74.5–127.0]	109 [82.0–142.5]	138 [96.8–186.5]	136 [88.0–172.0]	144 [97.5–189.0]	132 [100.0–194.0]
GFR	72.8 [47.61–97.16]	65.4 [47.11–84.31]	50.6 [37.67–76.36]	50.7 [36.83–83.60]	51.5 [35.92–74.75]	50.5 [35.46–74.96]
INR	1.1 [1.00–1.30]	1.6 [1.40–1.90]	1.5 [1.2–1.7]	1.3 [1.1–1.58]	1.2 [1.10–1.40]	1.2 [1.10–1.30]
MELD	10 [7.0–14.8]	17 [13.0–24.8}	20 [15.0–28.0]	22 [12.0–28.8]	17 [12.0–22.0]	16 [12.0–21.0]
CTP	6 [5.0–7.0]	7 [6.0–8.0]	7 [6.0–8.0]	7 [6.0–8.0]	6 [5.0–7.0]	6 [5.0–7.0]
ECMO flow [L/m^2^]	-	2.5 [2.17–2.75]	2.5 [2.25–2.79]	2.5 [2.18–2.77]	2.5 [2.01–2.79]	2.4 [1.81–2.87]

## Data Availability

The data presented in this study are available on request from the corresponding author (privacy reasons).
